# Outcome of osteofibrous dysplasia-like versus classic adamantinoma of long bones: a single-institution experience

**DOI:** 10.1186/s13018-020-01769-5

**Published:** 2020-07-16

**Authors:** Zhiping Deng, Lihua Gong, Qing Zhang, Lin Hao, Yi Ding, Xiaohui Niu

**Affiliations:** 1 Department of Orthopaedic Oncology Surgery, Beijing Jishuitan Hospital, Peking University, Number 31, Xinjiekoudongjie Street, Xicheng District, Beijing, 100035 China; 2 Department of Pathology, Beijing Jishuitan Hospital, Peking University, Beijing, China

**Keywords:** Osteofibrous dysplasia-like adamantinoma, Adamantinoma, Bone sarcoma, Neoplasm, Oncology

## Abstract

**Background:**

The clinical and molecular characteristics of osteofibrous dysplasia (OFD)-like adamantinoma (AD) differ from those of classic AD. Most reports about OFD-like AD are case reports or small case series. More cases from different centers are still warranted.

**Methods:**

The orthopedic oncology database of our institution was searched to identify patients with AD. The cases of OFD-like and classic AD of the long bones were retrospectively analyzed. Between December 1999 and August 2016, 23 patients were treated for AD, comprising seven with OFD-like AD and 16 with classic AD. The outcomes were compared between AD subtypes.

**Results:**

In the OFD-like AD group, four lesions were treated with extensive curettage, while three were treated with wide resection. The median follow-up duration in the OFD-like AD group was 66 months (range 43–131 months). At the end of follow-up, there was only one case of local recurrence (LR) in the OFD-like AD group, giving a LR rate of 14.3% (1/7). No distant metastasis or progression to classic AD was detected in the OFD-like AD group. In the classic AD group, the treatments were below-the-knee amputation in one patient with extensive tibial and fibular lesions, curettage with a bone graft in one patient who was diagnosed with OFD based on a core needle biopsy, hemi-cortical excision and reconstruction in two patients, and segmental resection and reconstruction in 12 patients. At the end of follow-up, there were three cases of LR in the classic AD group, giving a LR rate of 18.8% (3/16); two patients developed lung metastasis after LR and died of the disease at 88 and 126 months after the first surgery in our hospital, respectively. The classic AD group had a metastatic rate of 12.5% (2/16), a final limb salvage rate of 75%, and estimated 5- and 10-year survival rates of 88.9% and 77.1%, respectively.

**Conclusions:**

OFD-like AD has a better outcome than classic AD. For OFD-like AD, extensive curettage is suggested if the tumor extent allows. For classic AD, aggressive resection with wide margins is essential to achieve local control. A long-term follow-up is necessary due to the possibility of late complications.

## Highlights

- OFD-like AD has a better outcome than classic AD.
- For OFD-like AD, extensive curettage is suggested.
- For classic AD, aggressive resection with wide margins is essential.
- A long-term follow-up is necessary due to the possibility of late complications.

## Introduction

Adamantinoma (AD) of the long bones is an extremely rare bone tumor that was named because of its close morphological resemblance to AD of the jaw [1]. It can be classified into classic and OFD-like AD subtypes. In most cases, AD is localized to the tibia, especially the median third of the diaphysis, but it has also been described in the fibula, ulna, humerus, femur, and feet [2–6]. AD is located at the cortex and can invade the marrow space. Osteofibrous dysplasia (OFD) has similar imaging characteristics to AD and shows the fibro-osseous tissue in histology, which is thought to have the potential to progress to AD [7, 8]. OFD-like AD is a subtype of AD that is characterized by a uniform predominance of the OFD-like fibro-osseous tissue and a lack of conspicuous nests and masses of epithelial cells on histopathology [9]. In classic AD, the epithelial nest must be found at the background of the fibro-osseous tissue. The molecular characteristics of OFD-like AD reportedly vary from those of classic AD [10]. There are also differences between OFD-like AD and classic AD regarding the clinical characteristics and prognosis. However, most reports about OFD-like AD are case reports or small case series [9, 11–14]. Thus, more cases from different centers are still warranted. We reviewed the database of our institution and followed-up all cases of OFD-like AD and classic AD to evaluate and compare the treatment outcomes of these two subtypes.

## Material and methods

The present study was a retrospective analysis performed to evaluate the treatment outcomes of OFD-like AD compared with classic AD. Data were collected from the orthopedic oncology database of our institution. The inclusion criterion was a pathological diagnosis of OFD-like AD or classic AD of the long bones. The pathology specimens from all cases identified in the database search were reevaluated by an orthopedic pathologist. The diagnosis of AD subtype was confirmed by hematoxylin-eosin and immunohistochemical staining. Twenty-three patients who were treated between December 1999 and August 2016 were enrolled in the study. The final diagnosis was OFD-like AD in seven patients, and classic AD in 16.

After routine plain radiography, the patients underwent CT or MRI to evaluate the intraosseous extent of the tumor and soft tissue mass after referral to our hospital. CT of the chest and bone scan was conducted to evaluate the distant metastasis status. Lesion specimens underwent histological examination, including hematoxylin-eosin staining and immunohistochemical staining for cytokeratin expression.

**Fig. 1:**
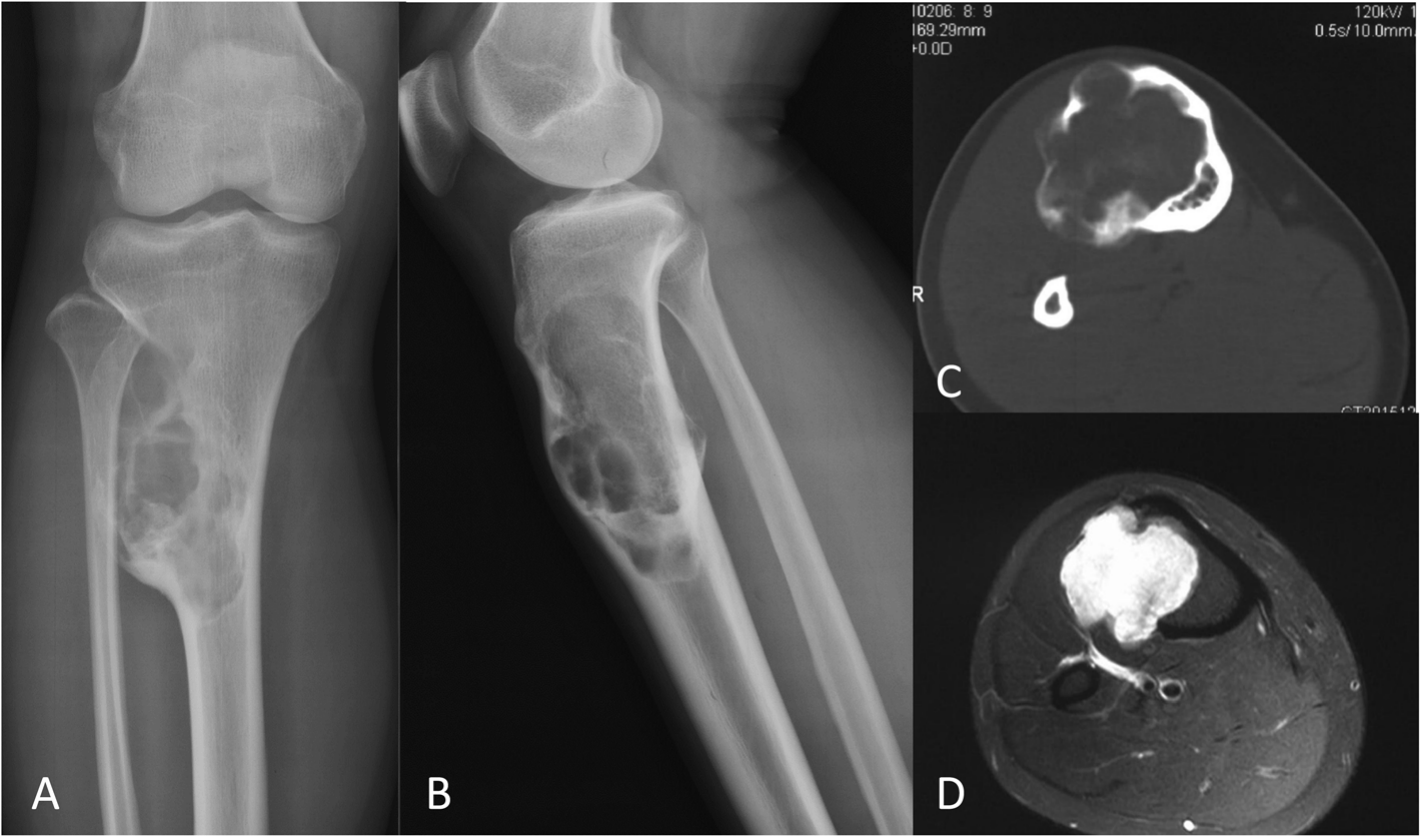
Representative images of osteofibrous dysplasia-like adamantinoma. Radiographs showing **a** anterior-posterior and **b** lateral views of the tumor in the tibial shaft. **c** CT image showing the extent of the tumor in the cortex. **d** MRI showing the extent of the tumor

**Fig. 2:**
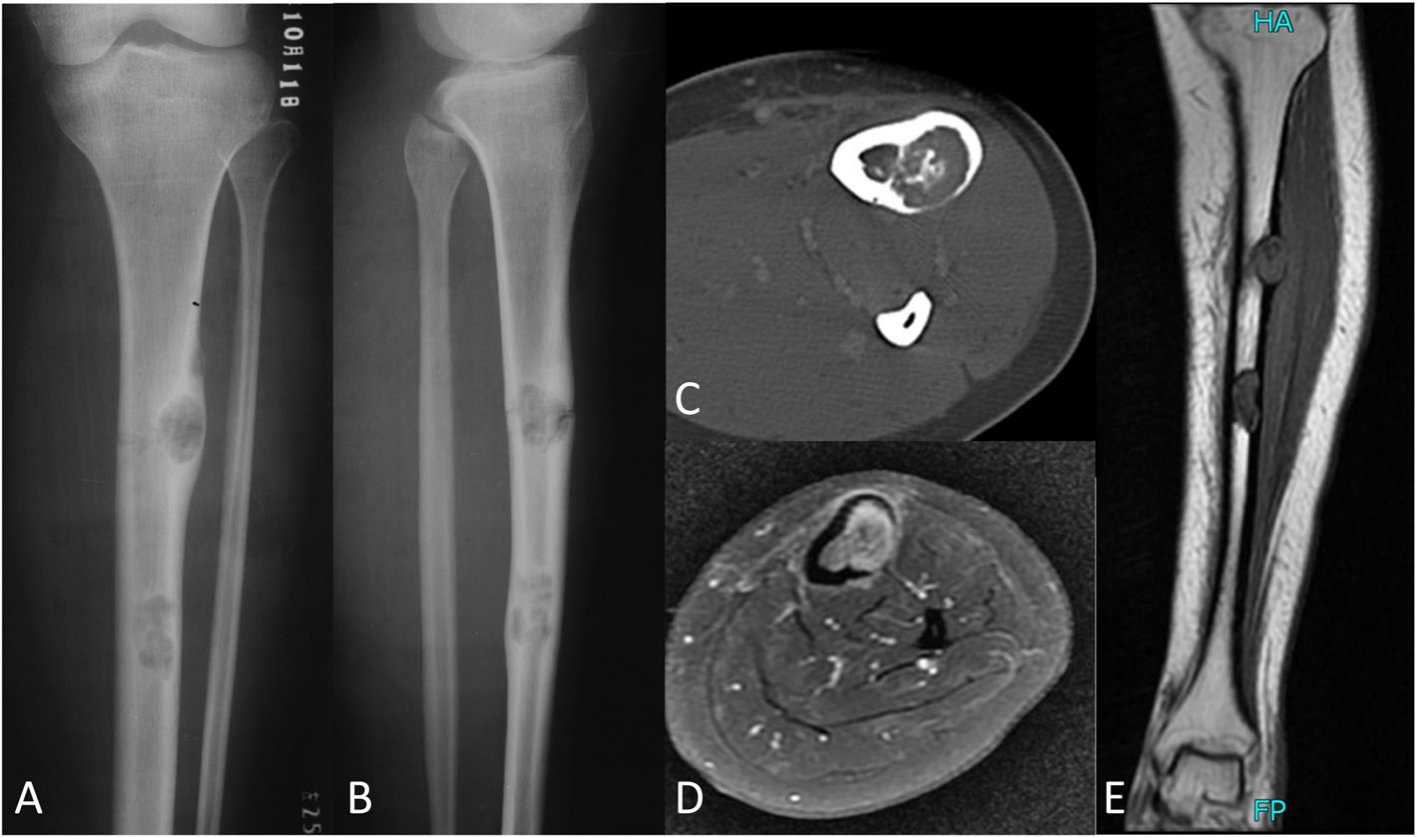
Representative images of classic adamantinoma. Radiographs showing **a** anterior-posterior and **b** lateral views of two lesions in the tibial shaft. **c** CT image showing the extent of the tumor in the cortex. **d**, **e** MR images showing the extent of the tumor

OFD-like and classic AD showed similar imaging characteristics (Figs. 1 and 2). The tumor was located in the cortex of the tibia or fibula, which showed expansile lytic lesions with varying degrees of osteolysis and osteosclerosis. CT showed cortical destruction, with cysts separated by sclerotic bony septa. MRI revealed different tumor foci with high signal intensities on T2-weighted images and T1-weighted contrast-enhanced images. Imaging showed whether the lesions were fibro-osseous, but neither CT nor MRI was able to differentiate between OFD-like and classic AD.

**Fig. 3:**
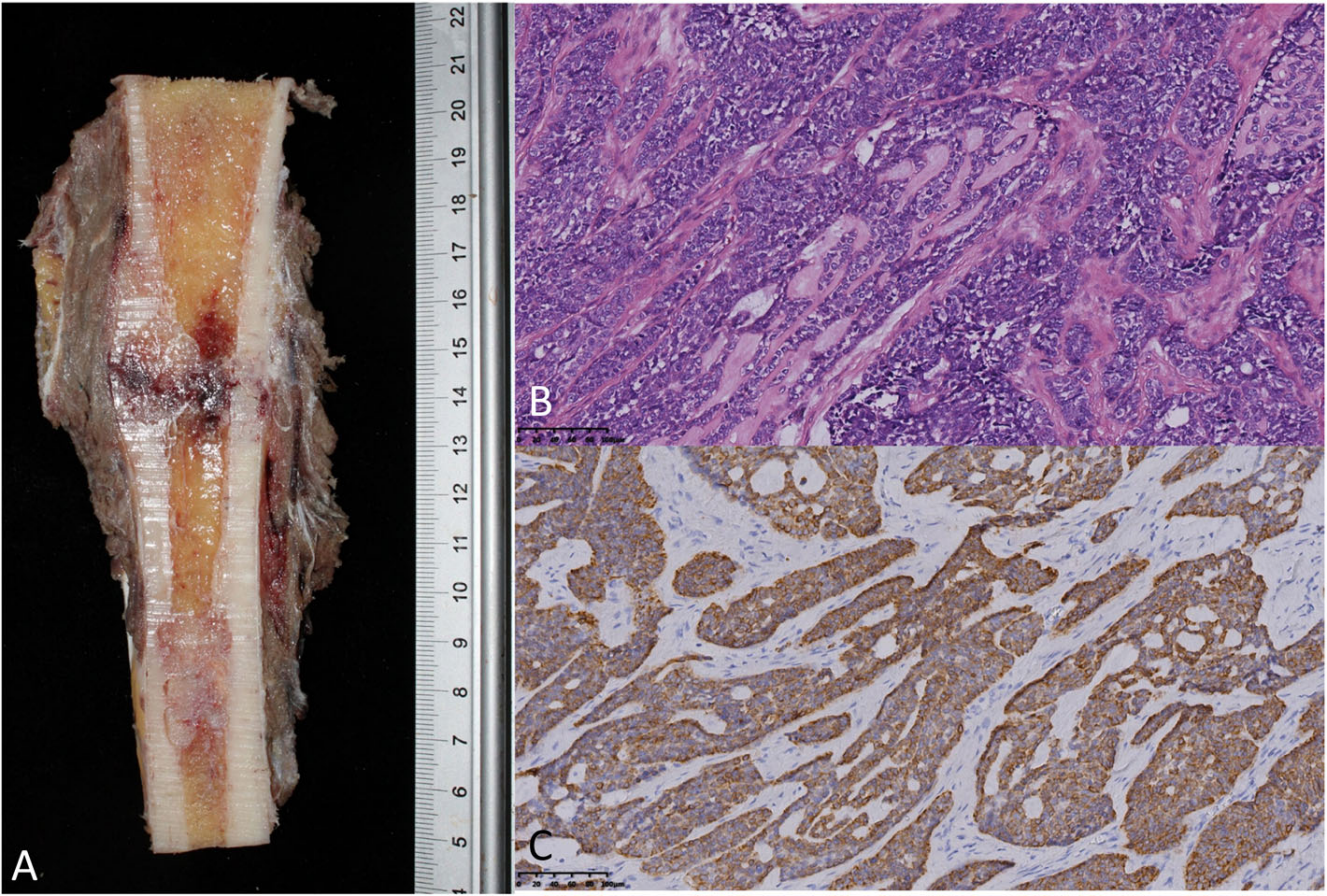
Histological images of classic adamantinoma. **a** Macroscopic section of the specimen in the coronal plane. **b**, **c** Hematoxylin-eosin staining (× 200 magnification) and immunohistochemical staining (cytokeratin+)

**Fig. 4:**
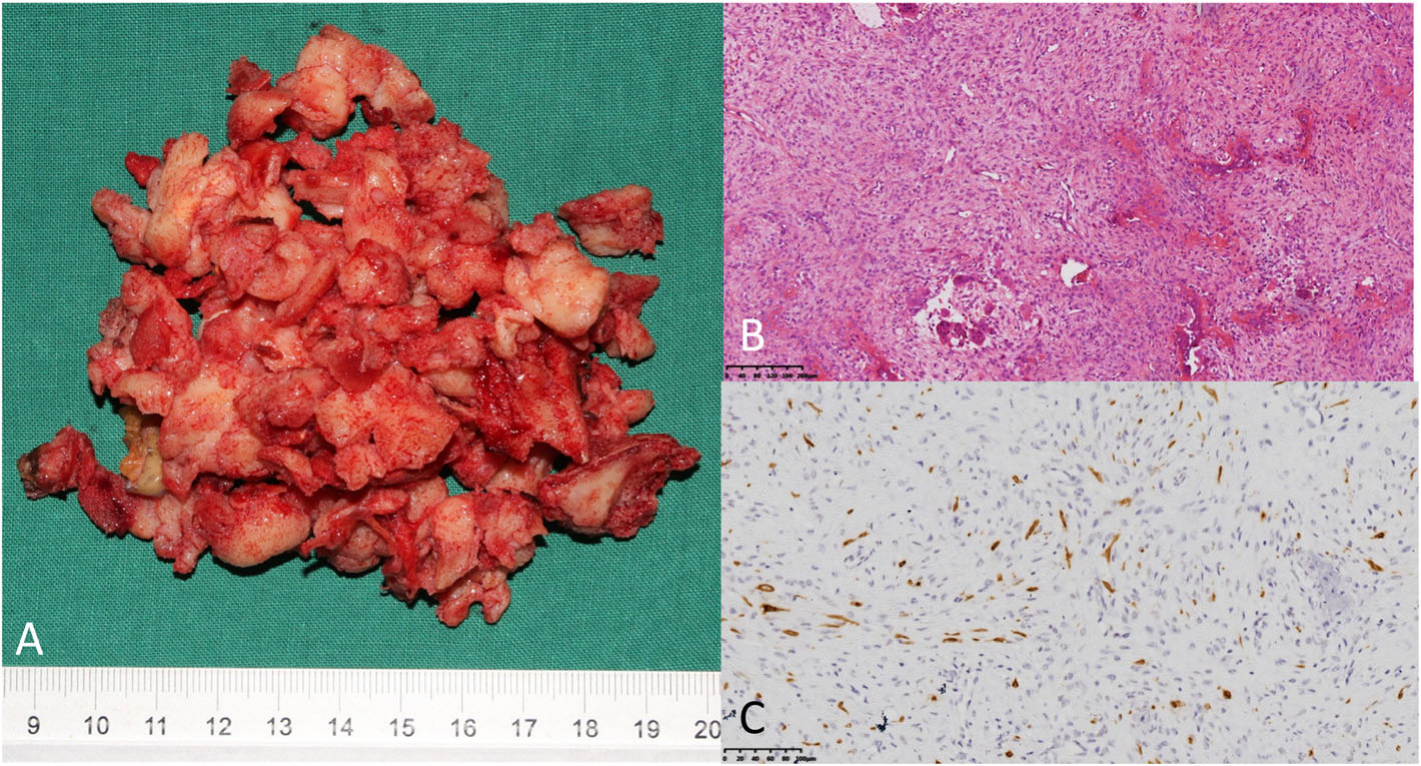
Histological images of osteofibrous dysplasia-like adamantinoma. **a** Specimen after curettage. **b**, **c** Hematoxylin-eosin staining (× 100 magnification) and immunohistochemical staining (cytokeratin+)

On histologic examination, both OFD-like and classic AD showed epithelial and osteofibrous components, but in different proportions. In classic AD, the epithelial component was prominent and was tubular, squamous, basaloid, or spindle-shaped with a minor proportion of OFD-like areas (Fig. 3). In OFD-like AD, there were small clusters of epithelial cells that were difficult to distinguish by light microscopy and showed cytokeratin expression on immunohistochemical staining (Fig. 4). The predominance of the osteofibrous tissue containing small clusters of epithelial cells and cytokeratin-positive staining was the diagnosis criteria for OFD-like AD which could differentiate it from classic AD.

For seven cases of OFD-like AD, the diagnosis was confirmed by reevaluating pathology in the two patients with the previous curettage and via biopsy in two other cases; the remaining three cases were initially diagnosed via biopsy as OFD and subsequently diagnosed as OFD-like AD on postoperative pathology. For classic AD, the diagnosis was confirmed by reevaluating pathology with the previous curettage in four patients, and via biopsy in eleven cases, and one case was diagnosed as OFD via biopsy and diagnosed as classic AD by postoperative histology.

After surgery, the local sites were surveilled by radiography and ultrasound, while distant locations were surveilled by CT of the chest. The bony local recurrence (LR) could be detected by radiography, and soft tissue LR might be detected by ultrasound. If LR was suspected through radiography and ultrasound, then CT or MRI was conducted and biopsy was performed for confirmation. The surveillance was performed every 3–4 months for the first 2 years postoperatively and then every 6–12 months.

Statistical analyses were performed using SPSS Statistics for Windows software (version 19.0; IBM Corp, Armonk, NY). The Kaplan-Meier method was used to detect the estimated survival rates.

## Results

### Outcome of the OFD-like AD group

**Fig. 5:**
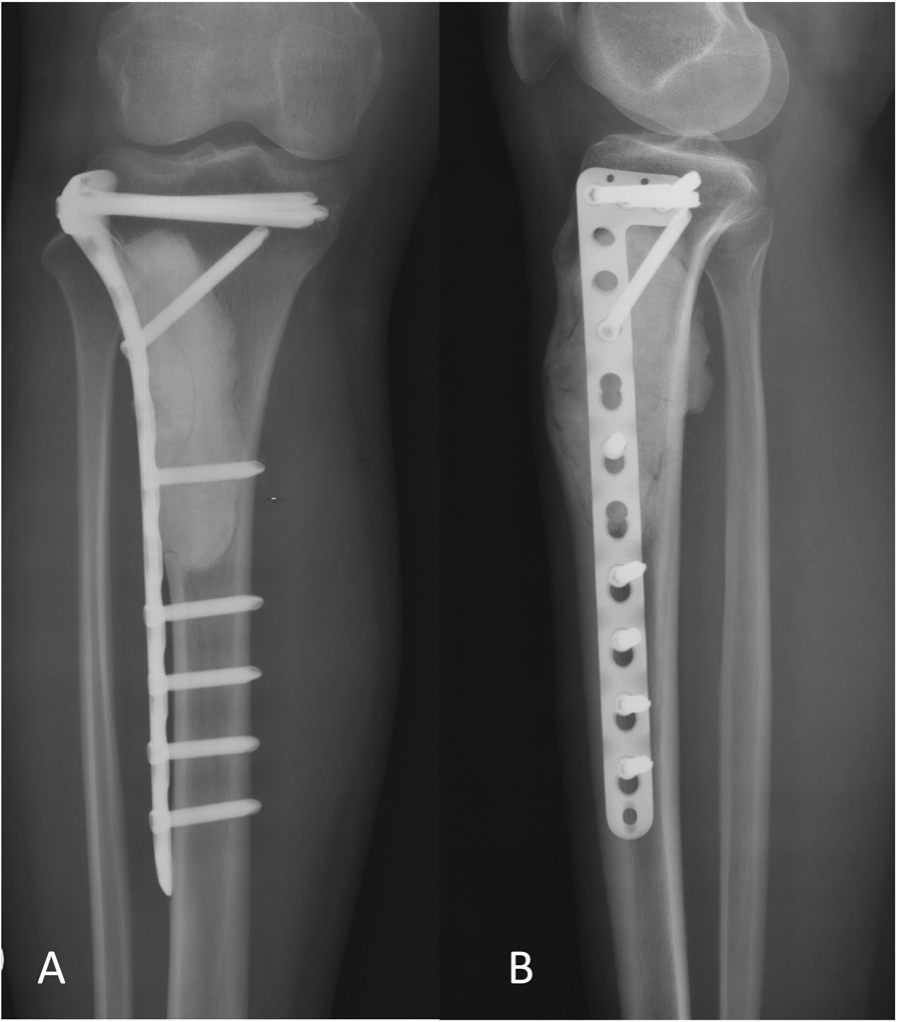
Surveillance imaging for osteofibrous dysplasia-like adamantinoma. **a**. Anterior-posterior and b. lateral views of the tibia at 1 year postoperatively

The OFD-like AD group had a median age of 17 years (range 14–22 years) and comprised three males and four females. Three patients had one lesion in the tibia, two had multiple lesions in the tibia, and two had lesions in both the tibia and fibula. Two patients had undergone previous curettage in other hospitals, while five had new lesions with no previous surgery. The treatment was extensive curettage (intralesional margins) in four cases and wide resection (wide margins) in three cases due to the extent of the lesions (Table 1). The extensive curettage performed in our hospital included opening a sufficiently large bone window, removing the tumor with a curette, expanding the margins with a high-speed bur, and inactivating the walls with argon beam ablation. The median follow-up duration was 66 months (range 43–131 months). At the end of follow-up, there was only one case of LR, giving a LR rate of 14.3% (1/7). The one patient with LR had previously undergone curettage and bone grafting along almost the whole length of the tibia in another hospital and had an osteolytic lesion in the distal part of the tibia at the time of referral to our hospital. Extensive curettage and cementation were performed in the distal part of the tibia. Six months later, another osteolytic lesion was detected in the proximal part of the tibia, and curettage was performed again. At the end of follow-up, all seven patients were alive (Fig. 5). No distant metastasis or progression to classic AD was detected. One patient who had undergone resection developed an uncontrollable allograft infection and finally underwent below-the-knee amputation.

**Table 1:** Clinical features, treatment, outcome, and follow-up of seven patients with osteofibrous dysplasia-like adamantinoma

Patient number	Age gender	Initial localization lesion	Previous surgery	Initial diagnosis	Initial treatment	Surgical margins	Final diagnosis	LR	Other treatment	Outcome	FU months
1	14/M	Tibia, single	None	OFD	Curettage +bone graft	Intralesional	OFD-like AD			CDF	131
2	19/M	Tibia, single	None	OFD-like AD	Resection +Allograft	Wide	OFD-like AD			CDF	69
3	15/M	Tibia and fibula	Curettage	OFD-like AD	Resection +Allograft, fibula resection	Wide	OFD-like AD			CDF	86
4	17/F	Tibia, multiple	Curettage	OFD-like AD	Curettage +cementation	Intralesional	OFD-like AD	6 months	Curettage +cementation after LR	NED	65
5	15/F	Tibia and fibula	None	OFD-like AD	Resection +Allograft, fibula resection	Wide	OFD-like AD		24 months, amputation after allograft infection	CDF	66
6	22/F	Tibia, single	None	OFD	Curettage +cementation	Intralesional	OFD-like AD			CDF	50
7	17/F	Tibia,multiple	None	OFD	Curettage +bone graft	Intralesional	OFD-like AD			CDF	43

*CDF* continued to be disease-free, *F* female, *FU* follow-up, *LR* local recurrence, *M* male, *NED* no evidence of disease

### Outcome of the classic AD group

**Fig. 6:**
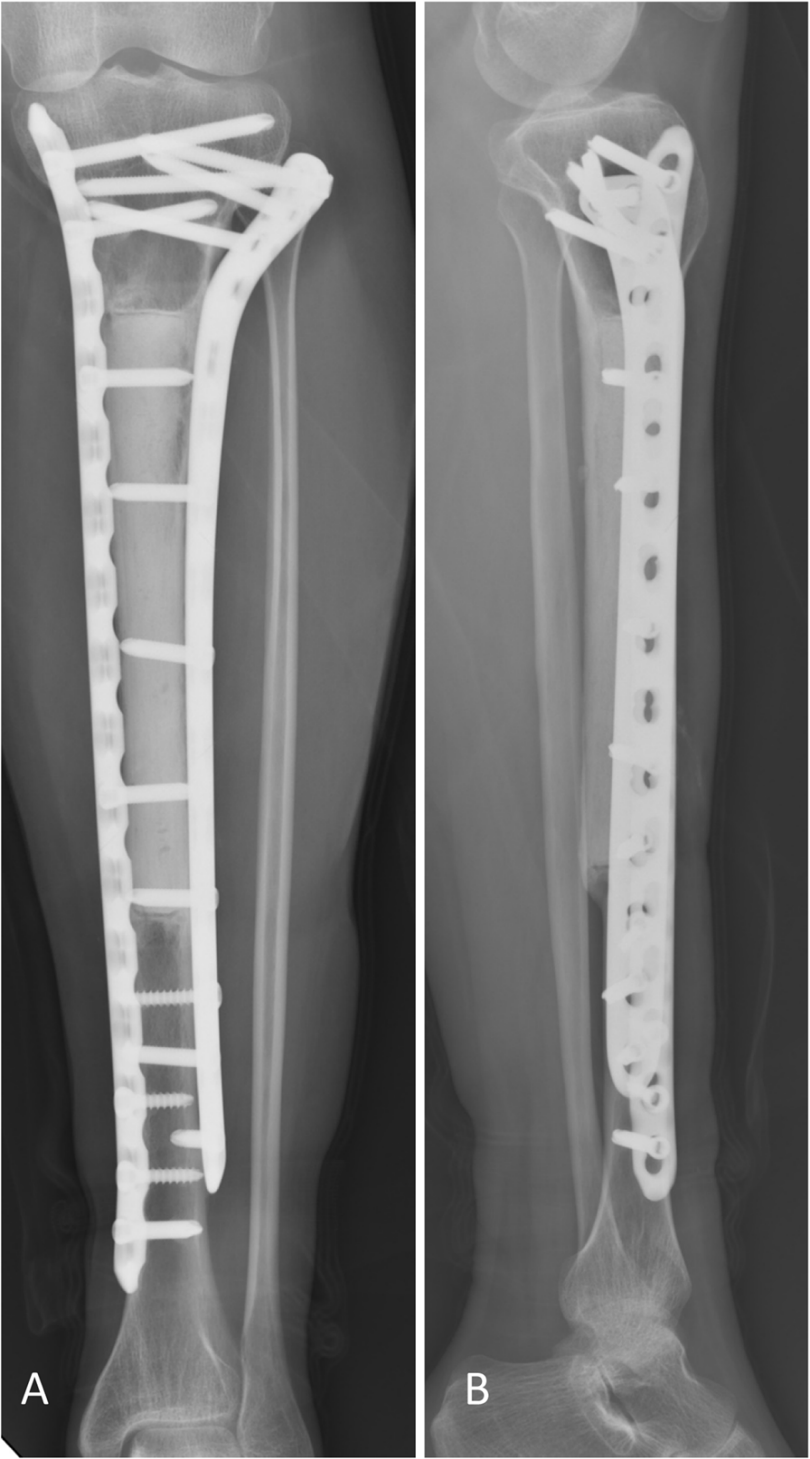
Surveillance imaging for classic adamantinoma. **a** Anterior-posterior and **b** lateral views of the tibia at 7 years postoperatively

**Fig. 7:**
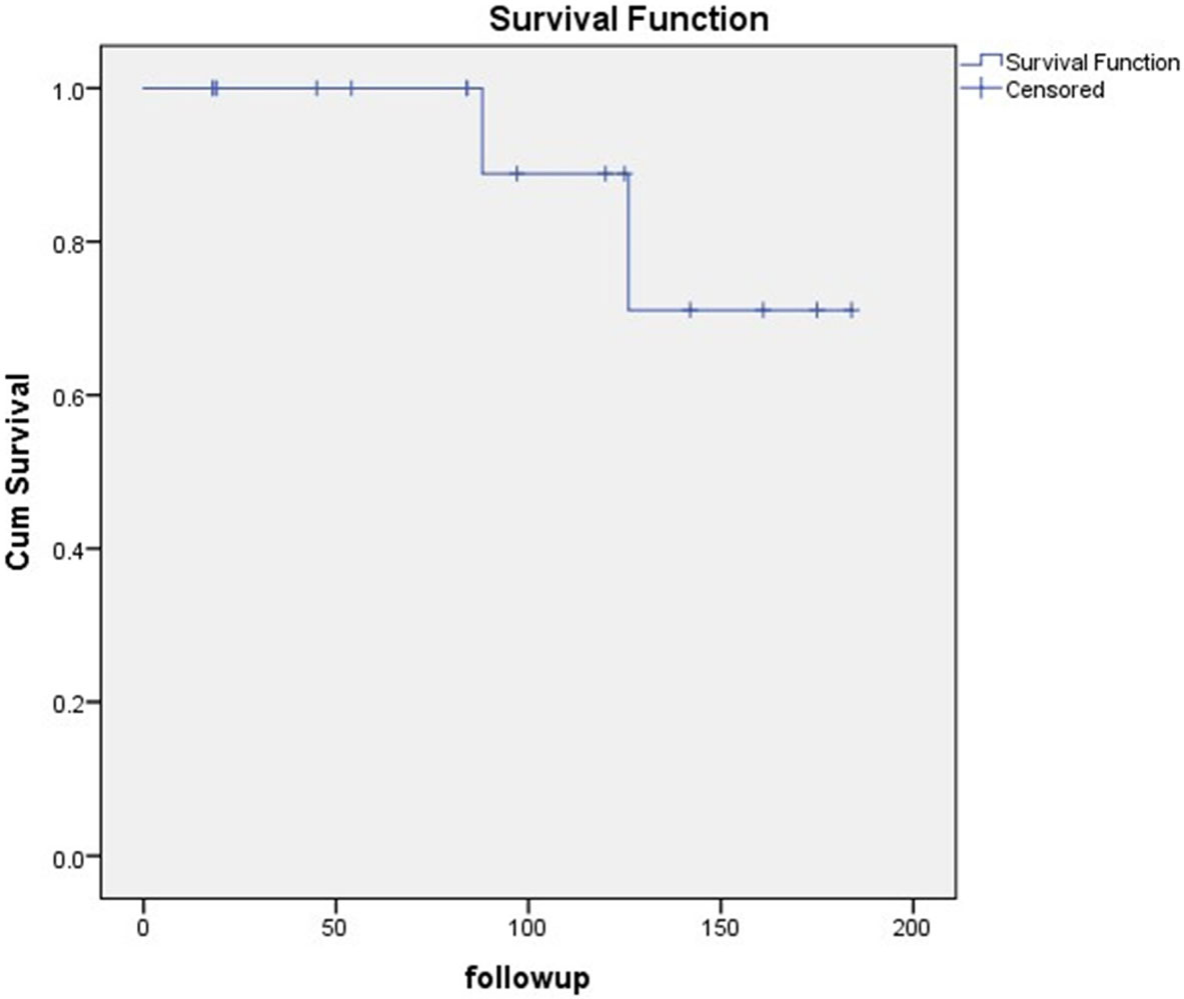
Kaplan-Meier overall survival curve of the 16 patients with classic adamantinoma

The classic AD group had a median age of 29 years (range 16–54 years) and comprised eight males and eight females. Seven patients had one lesion in the tibia, four had multiple lesions in the tibia, four had lesions in both the tibia and fibula, and one had one lesion in the fibula. Seven patients had undergone previous curettage in other hospitals and were referred to our hospital for lesion recurrence, while 9 patients had no previous history of surgery. The treatment was amputation (wide margins) in one patient with the extensive tibia and fibular lesions, curettage with bone grafting (intralesional margins) in one patient with a core needle biopsy diagnosis of OFD, hemi-cortical excision (marginal margins) and reconstruction in two patients, and segmental resection and reconstruction (10 wide margins and two marginal margins) in 12 patients (Table 2). One patient with a fibular lesion developed another lesion in the tibia at 7 years after the first surgery and underwent resection of the tibia. The median follow-up duration was 93 months (range 18–184 months) (Fig. 6). At the end of follow-up, there were three cases of LR (one with intralesional margins, one with marginal margins, and one with wide margins) that occurred 9, 19, and 84 months postoperatively, respectively. The LR rate was 18.8% (3/16). The LR rate was 40% (2/5) in the patients with inadequate margins (intralesional and marginal margins), and 9.1% (1/11) in those with adequate margins (wide margins). Three patients with LR were treated via amputation. Two patients developed lung metastasis after LR and died of the disease at 88 and 126 months, respectively, after the first surgery in our hospital. The metastatic rate was 12.5% (2/16). The final limb salvage rate was 75% (12/16). Previous surgery was a predisposing factor for the development of LR and metastasis. In the patients who had undergone previous surgery, the LR rate was 42.9% (3/7), and the metastasis rate was 28.6% (2/7). In the patients who had not undergone previous surgery, the LR rate was 0% (0/9), and the metastasis rate was 0% (0/9). The estimated 5- and 10-year survival rates for classic AD were 88.9% and 77.1%, respectively (Fig. 7).

**Table 2:** Clinical features, treatment, outcome, and follow-up of 16 patients with classic adamantinoma

Patient number	Age gender	Initial localization lesion	Previous surgery	Initial diagnosis	Initial treatment	Surgical margins	Final diagnosis	LR	Distant metastasis	Other treatment	Outcome	FU months
1	30/M	Tibia, multiple	None	AD	Resection +Recycled bone	Wide	AD				CDF	184
2	19/F	Tibia and fibula	Curettage	AD	Resection +Allograft, fibular resection	Wide	AD				CDF	161
3	46/M	Tibia, single	Curettage	AD	Resection +Allograft	Wide	AD				CDF	18
4	17/F	Tibia and fibula	None	AD	Resection +Allograft, fibular resection	Wide	AD				CDF	18
5	42/F	Tibia, single	None	AD	Resection +Allograft	Wide	AD				CDF	175
6	27/M	Tibia, single	Curettage	AD	Resection +Allograft	Wide	AD	84 months	106 m, lung	Amputation after LR	DOD	126
7	24/F	Tibia, single	None	AD	Cortical resection + Allograft	Marginal	AD				CDF	19
8	33/M	Tibia, single	None	AD	Cortical resection + Allograft	Marginal	AD				CDF	97
9	23/M	Tibia and fibula	Curettage	OFD	Curettage + Bone grafting	Intralesional	AD	9 months		Amputation after LR	NED	142
10	38/F	Tibia, single	Curettage	AD	Resection +Allograft	Wide	AD				CDF	125
11	33/M	Tibia,multiple	None	AD	Resection +Allograft	Marginal	AD				CDF	84
12	16/F	Fibula, single	None	AD	Resection	Wide	AD			84 m, new developed tibia lesion resection	NED	120
13	36/M	Tibia,multiple	Curettage	AD	Resection +Allograft	Marginal	AD	19 months	20 m, lung	Amputation after LR	DOD	88
14	20/F	Tibia,multiple	None	AD	Resection +Allograft	Wide	AD				CDF	84
15	18/M	Tibia and fibula	None	AD	BKA	Wide	AD				CDF	54
16	54/F	Tibia, single	Curettage	AD	Resection +Prosthesis	Wide	AD				CDF	45

*BKA* below the knee amputation, *CDF* continued to be disease-free, *DOD* dead of disease, *F* female, *FU* follow-up, *LR* local recurrence, *Lung* lung metastasis, *M* male, *NED* no evidence of disease

At the end of the follow-up, all the OFD-like AD patients were alive. The LR rate was 14.3%, and no distant metastasis or progression to classic AD was detected. In classic AD group, the LR rate was 18.8% and the distant metastatic rate was 12.5%. Two classic AD patients died of the disease. The outcome of OFD-like AD was better than the classic AD.

## Discussion

AD of the long bones is very rare and is a low-grade, primary malignant bone tumor composed of epithelial cells in a fibrous or osteofibrous stroma [15]. AD has a wide range of histological types, including classic and OFD-like subtypes [14]. A recently reported new subtype called dedifferentiated AD has a high-grade sarcoma component in a background of epithelial and osteofibrous cells [16–18]. The proportions of epithelial and osteofibrous components are used to differentiate OFD-like AD and classic AD. In OFD-like AD, only small clusters of epithelial cells are detected in a background of the osteofibrous tissue [14]. Therefore, a wide sampling and immunohistochemistry are crucial for the detection of these clusters of epithelial cells in OFD-like AD. If no epithelial cells are found, the lesion may be OFD, as OFD has similar imaging characteristics to OFD-like AD. In the current study, three OFD-like AD lesions were misdiagnosed as OFD at biopsy, probably because the biopsy samples were not large enough to find the clusters of epithelial cells. Thus, specimens from lesions diagnosed as OFD on histology should undergo immunohistochemical staining for cytokeratin to detect epithelial cells. In classic AD, there are different histological patterns, including basaloid, spindle-shaped, tubular, and squamous. However, all of these patterns have nests of epithelial cells on hematoxylin-eosin staining, which distinguishes these classic AD lesions from OFD-like AD [14].

Because of the radiologic similarities in OFD, OFD-like AD, and classic AD lesions, the relationship between these lesions has been discussed extensively. One study reported three cases of OFD that progressed to OFD-like AD [7], while another study reported two patients with presumed OFD-like AD who were diagnosed with classic AD at the time of LR [14]. This study supports the hypothesis that OFD-like AD may be a precursor lesion of classic AD [14]. Previous case reports have reported progression from OFD-like AD to classic AD [19] and possible progression from OFD to classic AD after LR [8]. In contrast, a recent study reported no progression from OFD or OFD-like AD to classic AD during a long-term follow-up of 42 patients with OFD, 10 with OFD-like AD, and 21 with classic AD [9]; however, six patients who were initially diagnosed with OFD were subsequently found to have OFD-like AD [9]. The authors highlighted the importance of imaging in the diagnosis of AD, as biopsy alone can result in sampling errors [9]. Similarly, three patients in the present study were initially diagnosed with OFD, but were postoperatively diagnosed with OFD-like AD. The present study found no evidence of progression from OFD-like AD to classic AD. More cases are still needed to determine the relationships between OFD, OFD-like AD, and classic AD.

Although OFD-like AD is a subtype of AD, the limited literature reports a benign but aggressive course. One study reported that two of seven patients with OFD-like AD who underwent surgical intervention developed LR, but no distant metastasis [9]. Another study that included five patients with OFD-like AD treated with curettage reported three cases of LR, but no metastasis or progression to classic AD [20]; two of the three patients with LR had pathological fractures, which might negatively affect local control [20]. Another three isolated case reports of OFD-like AD have reported no distant metastasis [11–13]. In the current study, the incidence of LR of OFD-like AD was 14.3%, and there was no metastasis detected. Classic AD is a low-grade tumor with a reported distant metastasis rate of 20–30%, and a reported 5-year survival rate of 92–95.5% [9, 21–23]. The present study showed a similar survival rate for classic AD (88.9%), but a slightly lower distant metastasis rate (12.5%), as our follow-up duration was not as long as in other studies. The size of the surgical margins is strongly related to the LR rate [23]. In the current study, the LR rate was 9.1% in the patients with adequate surgical margins, and 40% in those with inadequate margins; although this difference was marked, it was not statistically significant due to the small number of patients. Another factor that contributed to LR and metastasis in the present study was previous surgery in a non-sarcoma center. This previous surgery was usually performed by a general orthopedic surgeon, and extensive curettage via methods such as high-speed burring or argon beam ablation was not routinely performed. Furthermore, there is a risk of contamination of the soft tissue adjacent to the affected bone. Therefore, for patients with a history of previous curettage, the surrounding soft tissue should also be removed to attain wide margins and decrease the LR rate.

The current study highlights the different outcomes of OFD-like AD and classic AD. A recent study also revealed a molecular difference between these two subtypes [10]. Hierarchical clustering analysis of RNA-Seq data can be used to distinguish the two subtypes. DAVID Gene Ontology analysis of differentially expressed genes identified different altered pathways in the two subtypes of AD [10]. As OFD-like and classic AD have clinical and molecular differences, the two subtypes should be treated differently. Classic AD is a low-grade tumor with metastatic potential that requires aggressive surgery with wide margins, as it is not sensitive to chemotherapy and radiotherapy. In the literature and in the current study, OFD-like AD followed a benign but aggressive course [9, 11–13, 20]. There are some reports of LR of OFD-like AD, but no reports of distant metastasis. The surgery treatment modality for OFD-like AD should be cautiously selected [9]. In the current study, one patient of OFD-like AD with multiple tibial lesions treated with resection and allograft reconstruction developed a severe infection and had to undergo amputation. For lesions diagnosed as OFD-like AD, extensive curettage should be considered when the extent of the tumor permits such curettage to avoid severe complications since the oncological outcome is acceptable for intralesional treatment. There was no distant metastasis and only one LR (14.3%) of OFD-like AD in the current study. However, long-term follow-up is still warranted due to the possibility of progression from OFD-like to classic AD [14].

The present study had some limitations. First, the sample size was relatively small. Thus, although inadequate margins and a history of previous surgery seemed to contribute to LR, the small number of cases meant that these findings failed to reach a statistical significance. However, a small sample size is a common problem in studies evaluating rare diseases. The small sample size in the present study reflects the rarity of OFD-like and classic AD, even in a center that specializes in treating the bone and soft tissue tumors. Second, this was a mono-institutional study, although all the procedures were performed by experienced surgeons in our department, the multi-centric study was still warranted.

The data of our study suggests that OFD-like AD has a better outcome than classic AD. For OFD-like AD, extensive curettage is suggested if the tumor extent allows this. For classic AD, aggressive surgery with wide margins is essential to achieve local control. A long-term follow-up is necessary due to the possibility of late complications.

## Data Availability

The datasets used and analyzed during the current study are available from the corresponding author on reasonable request.

## Abbreviations

AD: Adamantinoma
BKA: Below the knee amputation
CDF: Continued to be disease-free
DOD: Dead of disease
F: Female
FU: Follow-up
LR: Local recurrence
M: Male
NED: No evidence of disease
OFD: Osteofibrous dysplasia
